# Acute Effects of a Heat-Not-Burn Tobacco Product on Pulmonary Function

**DOI:** 10.3390/medicina56060292

**Published:** 2020-06-12

**Authors:** Athanasia Pataka, Seraphim Kotoulas, Evangelos Chatzopoulos, Ioanna Grigoriou, Konstantinos Sapalidis, Christoforos Kosmidis, Anastasios Vagionas, Εleni-Isidora Perdikouri, Konstantinos Drevelegas, Paul Zarogoulidis, Paraskevi Argyropoulou

**Affiliations:** 1 Respiratory Failure Unit, Respiratory Medicine, Medical School, G. Papanikolaou Hospital, Aristotle University of Thessaloniki, 541 24 Thessaloniki, Greece; patakath@yahoo.gr (A.P.); nmichalopoulos1@outlook.com (S.K.); isaackesisoglou@outlook.com (E.C.); stellalaskou@outlook.com (I.G.); zeinepmemet@outlook.com (P.A.); 2 3rd Department of Surgery, Medical School, AHEPA University Hospital, Aristotle University of Thessaloniki, 541 24 Thessaloniki, Greece; sapalidiskonstantinos@gmail.com (K.S.); dr.ckosmidis@gmail.com (C.K.); 3 Oncology Deaprtment, General Hospiatl of Kavala, 655 00 Kavala, Greece; tvagionas@yahoo.com; 4 Oncology Department, General Hospital of Volos, 382 22 Volos, Greece; eigrouse@yahoo.gr; 5 Radiology Department, Euromedica Private Diagnostic Laboratory, 541 24 Thessaloniki, Greece; kdrev@hotmail.com

**Keywords:** heated tobacco, IQOS, respiratory function, resistance, exhaled CO, acute effects

## Abstract

*Background and objectives*: During the last decade, conventional tobacco smoking is experiencing a decline and new smoking products have been introduced. IQOS (“I-Quit-Ordinary-Smoking”) is a type of “heat-not-burn” (HNB) tobacco product. The impact of IQOS on respiratory health is currently not defined. The objectives of this study were to evaluate the acute effects of IQOS on pulmonary function in non-smokers and current smokers. *Materials and Methods:* Fifty male healthy non-smokers and current smokers with no known co-morbidity underwent an exhaled CO measurement, oximetry (SaO2%), pulmonary function tests (flows, volumes and diffusion capacity), and a measurement of respiratory resistances with an impulse oscillometry system (IOS) before and immediately after IQOS use. *Results:* In the whole group of 50 participants, SaO2%, forced expiratory flow at 25% and 50% of vital capacity (FEF 25%, FEF 50%, respectively), peak expiratory flow (PEF), and diffusion lung capacity for carbon monoxide/VA (KCO) decreased significantly after IQOS use, whereas exhaled CO and airway resistance (R5 Hz, R10 Hz, r15 Hz, R20 Hz, R25 Hz, R35 Hz) increased. When the groups of smokers and non-smokers were compared, in both groups (all males, 25 smokers and 25 non-smokers), exhaled CO increased and SaO2% decreased after IQOS use (*p* < 0.001). In the group of non-smokers, PEF (pre 8.22 ± 2.06 vs. post 7.5 ± 2.16, *p* = 0.001) and FEF 25% (pre 7.6 ± 1.89 vs. 7.14 ± 2.06, *p* = 0.009) decreased significantly; respiratory resistances R20 Hz (pre 0.34 ± 0.1 vs. post 0.36 ± 0.09, *p* = 0.09) and R25 Hz (pre 0.36 ± 0.1 vs. post 0.38 ± 0.09, *p* = 0.08) increased almost significantly. In smokers, PEF (pre 7.69 ± 2.26 vs. post 7.12 ± 2.03, *p* = 0.007) and expiratory reserve volume (ERV) (pre 1.57 ± 0.76 vs. post1.23 ± 0.48, *p* = 0.03) decreased and R35 Hz (pre 0.36 ± 0.11 vs. post 0.39 ± 0.11, *p* = 0.047) increased. The differences in the changes after the use of IQOS did not differ between groups. *Conclusions:* IQOS had an impact on exhaled CO, SaO2%, and airways function immediately after use. Even though these changes were rather small to be considered of major clinical importance, they should raise concerns regarding the long-term safety of this product. Further research is needed for the short- and long-term effects of IQOS, especially in patients with respiratory disease.

## 1. Introduction

Cigarette smoking is the leading cause of preventable diseases. In order to reduce the burden of smoking-related diseases, many countries have developed tobacco control strategies. During the last decade, conventional tobacco smoking is experiencing a decline and new smoking products such as electronic cigarettes (EC) and “heat-not-burn” (HNB) tobacco products have been introduced in the market all around the world. In conventional cigarettes (CC), the tobacco is burned (>600 °C), releasing smoke containing harmful chemicals [1]. CC smoking has long been strongly associated with the development of lung inflammation leading to respiratory disease as chronic obstructive pulmonary disease (COPD), asthma, interstitial lung disease, and lung cancer. Additionally, cigarette smoke alters lung immunity, increasing lung infections, and is associated with oxidative stress and cardiovascular diseases as myocardial infarction and stroke [2]. The tobacco industry has introduced the new tobacco products as “safer” alternatives to CC. Studies evaluating EC health effects and safety are still ongoing. EC vapor has been found to increase oxidative stress and inflammation, to impair pulmonary defenses and to have immunomodulatory effects similar to that of CC smoke [3]. It has been found that short-term use of EC acutely affects respiratory symptoms and impacts airways physiology in healthy never smokers, in smokers with no known co-morbidity, and in smokers with respiratory conditions as asthma and COPD [4,5,6].

The tobacco industry promotes tHNB products as “reduced risk”, as they claim that by reducing the temperature (<350 °C), they may generate lower levels of harmful chemicals (including CO) with reduced toxicity compared with CC smoke [7]. In a recent systematic review [8], 20 out of 31 studies for HNB products were performed from the tobacco industry. The lack of independent evidence to validate manufacturers’ data is a challenge. IQOS (I-Quit-Ordinary-Smoking) is a type of a HNB tobacco product manufactured by Philip Morris International (PMI) [7]. The effects of IQOS on pulmonary function, short- and long-term, have not been adequately studied yet. Previous studies have found that CC and EC smokers present significantly higher airway resistances compared with non-smokers after short-term use [4,5,6]. The aim of this study was to evaluate the acute effects of IQOS on the pulmonary function of non-smokers and current smokers with no known co-morbidities.

## 2. Materials and Methods

Our study included 50 adults (all men, 25 smokers and 25 non-smokers) recruited from a community setting. Current cigarette smokers were defined as those smoking ≥1 cigarettes during the past 30 days with a minimum 5 pack-years. Exclusion criteria included systematic use of IQOS or electronic cigarette (EC) or other tobacco products as cigars, pipe, and water pipe, among others, or marijuana, current use of any medication, acute illness during the previous 2 weeks, and any chronic and/or lung disease. All subjects were instructed not to smoke for at least 6 h prior to the examination. Exhaled CO measurement (piCO Smokerlyzer, Bedfont Scientific Ltd., Kent, UK) was performed before the examination and participants were included in the study if exhaled CO measurements were less than 3 parts-per-million. The local ethics committee provided ethics approval and each subject read and signed a written informed consent form. The study is registered in ClinicalTrials.gov: NCT03889990, NCT03995329.

All participants underwent initial measurements of pulmonary function tests (PFTs) (spirometry, static lung volumes and diffusion lung capacity for carbon monoxide) (MasterScreen PFT, Jaeger, Wurzburg, Germany). Forced vital capacity (FVC), forced expiratory volume in 1 s (FEV1), FEV1/FVC ratio, forced expiratory flow (FEF) at 25%, 50%, and 75% of vital capacity, peak expiratory flow (PEF), expiratory reserve volume (ERV), functional residual capacity (FRC), total lung capacity (TLC), residual volume (RV), and diffusion lung capacity for carbon monoxide (DLCO) were measured according to guidelines [9]. Total respiratory resistances including respiratory impedance at 5 Hz (Z5 Hz) and respiratory resistance at 5, 10, 20, 25, and 35 Hz (R5 Hz, R10 Hz, R20 Hz, R25 Hz, and R35 Hz, respectively) were assessed with an impulse oscillometry system (IOS) (Viasys Jaeger MasterScreen IOS system) [10]. Oxygen saturation (SaO2%) was measured by pulse oximetry.

The participants of both groups “smoked” the IQOS tobacco stick, with the mean nicotine content of 0.50 mg/stick, for up to 14 puffs (almost 5–6 min) [7]. For every participant, a new tobacco stick was used. IQOS includes a holder, a charger, and tobacco sticks (Heets). The tobacco stick is “heated” with an electronically controlled heating blade (<350 °C). Immediately after IQOS (5.6 ± 1.3 min), all participants were subjected again to the aforementioned measurements.

Statistical analysis was performed using the SPSS software (version-20 IBM-SPSS-statistical-software, Armonk, NY, USA). Sample size was calculated using the G*Power software (Die-Heinrich-Heine-Universität, Düsseldorf, Germany). The difference in the airway impedance (Z) before and after IQOS was used as the primary endpoint of the study. Since there were no studies examining the acute effect of IQOS in Z at the time of conducting our research, we calculated the sample size for each group of our study by considering statistical significant as a change of 0.025 kPa/L/s (pre = 0.37 ± 0.06 vs. post = 0.42 ± 0.07), which was the respective change in Z before and after a conventional tobacco cigarette in another study [11]. The expected effect size was calculated at 0.76, with the power at 0.95 and the level of statistical significance at 0.05, with the test being two-tailed and the total sample size being calculated at 25 participants for each group. Continuous variables were presented as mean ± SD and non-normally distributed data were expressed as median. Tests were two-tailed and *p* < 0.05 was accepted as statistically significant. In order to separate the parametric from the non-parametric variables in the normality tests, the Shapiro–Wilk test was performed. For independent samples, a T test and Mann–Whitney U test for parametric and non-parametric variables were respectively used to detect differences between two groups. A paired samples T test for the parametric or Wilcoxon signed ranks test for the non-parametric variables were used to detect differences before and after IQOS for each group.

## 3. Results

The basic characteristics of the participants are presented in Table 1. When the groups of smokers and non-smokers were compared, no significant differences were observed in the anthropometric measures (Table 1). The acute effects of IQOS on the respiratory function, exhaled CO, and SaO2% of all 50 participants are presented in Table 2.

In the whole group of 50 participants, SaO2%, forced expiratory flow at 25% and 50% of vital capacity (FEF 25%, FEF 50%, respectively), peak expiratory flow (PEF), and diffusion lung capacity for carbon monoxide/VA (KCO) decreased significantly after IQOS use, whereas exhaled CO and airway resistances (R5 Hz, R10 Hz, r15 Hz, R20 Hz, R25 Hz, R35 Hz) increased. Exhaled CO did not differ between groups before IQOS use as smokers were abstinent from CC for at least 6 h prior to the examination. Smokers had a median 13.5 (5–65) pack/years. Pre-IQOS exposure, respiratory function tests, and exhaled CO were similar in both groups of participants, apart from lower KCO in smokers (1.49 ± 0.27 vs. 1.74 ± 0.19, *p* = 0.001). In both groups, exhaled CO increased (*p* < 0.001) and SaO2% decreased after IQOS use (*p* < 0.001) (Table 2).

In the group of non-smokers, PEF (pre 8.22 ± 2.06 vs. post 7.5 ± 2.16, *p* = 0.001) and FEF 25% (pre 7.6 ± 1.89 vs.7.14 ± 2.06, *p* = 0.009) decreased significantly, whereas KCO decreased (pre 1.74 ± 0.19 vs. post 1.71 ± 0.2, *p* = 0.09) (Table 2); respiratory resistances R20 Hz (pre 0.34 ± 0.1 vs. post 0.36 ± 0.09, *p* = 0.09) and R25 Hz (pre 0.36 ± 0.1 vs. post 0.38 ± 0.09, *p* = 0.08) increased (Table 3). In smokers, PEF (pre 7.69 ± 2.26 vs. post 7.12 ± 2.03, *p* = 0.007) and ERV (pre 1.57 ± 0.76 vs. post 1.23 ± 0.48, *p* = 0.03) decreased (Table 2) and R35 Hz (pre 0.36 ± 0.11 vs. post 0.39 ± 0.11, *p* = 0.047) increased (Table 3).

The differences between the measurements before and immediately after IQOS use on respiratory function and airways resistance did not differ between groups (Table 2 and Table 3).

Additional analysis was conducted to control the effect of potential confounders as age and pack/years on the effect of IQOS use on pulmonary functions. No significant changes were observed on the differences between the measurements after adjusting for age and pack/years (Table 4).

## 4. Discussion

Our findings suggest that IQOS has an impact on airways function, exhaled CO, and SaO2% of both smokers and non-smokers with no reported co-morbidities. Even though these changes were rather small to be considered of major clinical importance, they should raise concerns regarding the long-term safety of this product on pulmonary function. There are data showing that the new nicotine products, as EC and HNB products, are used particularly by the young and never-smokers, representing a new way for nicotine addiction [12]. Additionally, the peak nicotine concentration ratio of IQOS was found to be comparable to that of CC [13]. EC vaping has been associated with pulmonary dysfunction expressed as increased airways resistance, oxidative stress [14], and respiratory symptoms as cough [4,5,6] in users with no co-morbidities and in patients with obstructive respiratory disease as asthma or COPD. However, in other studies, the use of ECs free of nicotine, was not found to significantly affect the pulmonary function of healthy or asthmatic patients [15].

In the current study, SaO2%, FEF 25%, FEF 50%, PEF, and KCO decreased significantly after IQOS use, whereas xhaled CO and airway resistances (R5 Hz, R10 Hz, R15 Hz, R20 Hz, R25 Hz, R35 Hz) increased in the whole group of 50 participants. When the groups of smokers and non-smokers were compared, PEF, FEF 25%, and KCO decreased in the group of non-smokers, whereas respiratory resistances R20 Hz and R25 Hz increased. In the group of smokers, PEF and ERV decreased, whereas R35 Hz increased. From previous studies on the short-term effects of CC on respiratory function, mid-to-small size pulmonary airways were firstly affected (FEF 25%, FEF 50%, FEF25–75%) in a dose-dependent manner, reflecting the considerable extent of subclinical inflammatory changes in the periphery of the bronchial tree, not only in the larger airways [11,16,17,18]. FEV1 changes were mainly related to mid and larger size airways and reflected the long-term rather than the acute effects of CC smoking, taking about a year to develop, depending on the duration and intensity of CC exposure [18,19]. The consumption of a single CC was found to increase immediately Z5 Hz and airway resistance (R 5 Hz, R 10 Hz, and R 20 Hz) [11]. Smoking abstinence or reduced exposure to CC smoke for three days was found to improve pulmonary function [18,19]. Moreover, a study on the effect of EC on the pulmonary function of smokers with mild asthma compared with smokers with no co-morbidities, immediately 15 and 30 min after vaping, found that Z5 Hz was more significantly increased in asthmatics and “healthy” smokers and that both total (R5 Hz) and large (R20 Hz) airway resistance was increased in both groups, indicating acute bronchoconstriction [6].

IQOS was developed by Philip Morris International (PMI) as a “reduced harm” alternative to conventional smoking in order to replace the nicotine uptake of CC [6]. Moazed et al. [20] assessed industry data on the pulmonary and immunosuppressive effects of HNB products in near-real-world conditions and additionally compared the participants with others who continued CC. Industry data did not show any improvement of lung function after three months of transition to IQOS compared with those who continued CC. FEV1/FVC increased significantly only in the smoking abstinence group [20]. A recent study, also found that IQOS has the same damaging effect on human airway epithelial and smooth muscle cells in vitro as the CC, contributing to altered mitochondrial function, airway inflammation, and remodeling [21].

The main conclusion of our study was that pulmonary function was immediately affected in all participants, both never-smokers and smokers, providing evidence that IQOS may impact airways function even after only 5 min of use. This could be attributed to bronchospasm, localized mucosal edema, or even secretions. In a study on the effects of EC vapor, nicotine, and cigarette smoke on the lung structure and blood vessel count, emphysematous changes were apparent in EC, nicotine-treated, and CC smoke-exposed rat lungs [22]. This was attributed to EC’s vapor particles with a diameter of 2.5 μm or less that may penetrate lung tissue and the blood stream leading to the negative effects on lung morphology [22]. Recent studies have found that the size of IQOS mainstream aerosol particles decreased as the temperature increased, from 100 nm at 37 °C to less than 20 nm at 300 °C [23].

Smoking status may be assessed either by measuring plasma or urine nicotine or cotinine, or more simply by measuring exhaled breath CO levels. The exhaled CO measurement is widely used to evaluate carboxyhaemoglobin (COHb) [24], in order to separate smokers from non-smokers [25] and to monitor the smoking habits of patients in smoking cessation programs. In a recent study [26], switching to either EC or IQOS for six months lead to a significant reduction in exhaled CO levels with the levels of exhaled CO being within the range of non-smokers. Exhaled CO levels did not differ at six months between EC or IQOS groups in terms of % COHb, but those switching to IQOS showed a significantly lower reduction in exhaled CO ppm values compared with EC [26]. However, there is also emerging research suggesting that IQOS produces high levels of carbonyls emissions [27]. In our study, the levels of exhaled CO were found within the CO exposure range observed in non-smokers, and were lower than the published levels of smokers [25], in accordance with the aforementioned study [26].

We want to mention that our study has some limitations. We evaluated only the acute effects of IQOS; it would be interesting to assess the duration of the effects and study IQOS users’ respiratory function in the long-term and to compare them with those of other tobacco products as conventional cigarettes. What is more, our sample size was relatively low, consisting only of healthy men. It would be interesting to investigate IQOS effects on the pulmonary function of women and also patients suffering from respiratory disease such as COPD or asthma.

In this study, we found that IQOS acutely alters lung function. Industry data suggest that IQOS reduces exposure to several harmful or potentially harmful constituents compared with CC. In a recent study by Philip Morris, switching from conventional cigarettes predominantly or completely to HNB products lead to higher FEV1% pred, decreased exhaled CO, and reduced exposure to carcinogens [28]. However, IQOS presented higher cytotoxic effects compared with EC, but lower compared with CC, in in vitro studies on bronchial epithelial cells [29]. Similarly to CC smoke and EC, IQOS also affected airway remodeling, and increased oxidative stress and inflammation [21]. IQOS was additionally found to contain higher levels of other substances whose toxicity and harm are not known yet [27]. Further research is needed for the long-term effects of this product on “chronic” users, with or without co-morbidities, as they would be exposed to it several times per day and for a longer duration.

## 5. Conclusions

This is an independent study on IQOS acute effects on the pulmonary function of non-smokers and smokers with no known co-morbidities. IQOS was found to have an impact on airways function, exhaled CO, and SaO2% of both smokers and non-smokers immediately after use. In all 50 participants, SaO2%, FEF 25%, FEF 50%, PEF, and KCO decreased significantly after IQOS use, whereas exhaled CO and airway resistances (R5 Hz, R10 Hz, R15 Hz, R20 Hz, R25 Hz, R35 Hz) significantly increased. More specifically, in the group of non-smokers, PEF, FEF 25%, and KCO decreased, while respiratory resistances increased (R20 Hz and R25 Hz). In the group of smokers, PEF and ERV decreased, whereas R35 Hz increased. Even though these changes were rather small to be considered of major clinical importance, they should raise concerns on the long-term safety of this product on lung function.

## Figures and Tables

**Table 1:** Basic characteristics of the participants.

	All Participants (*n* = 50)	Non Smokers(*n* = 25)	Smokers(*n* = 25)	*p*
**Age, years**	38.8 ± 11.9	37.4 ± 10.4	40.3 ± 13.2	0.4
**BMI, kg/m^2^**	25.02 ± 4.23	25.2 ± 4.7 7	24.8 ± 3.87 kg	0.7
**Pack/years**	0.5 (0–65)	0	13.5 (5–65)	<0.001
**Duration of cigarette use (years)**	0.2 (0–35)	0	11.2 (3–35)	<0.001

BMI: body mass index.

**Table 2:** Acute effects of IQOS on pulmonary function test of all participants, non-smokers and smokers—differences between the measurements before and immediately after IQOS use.

	All Participants (*n* = 50) *			Non Smokers(*n* = 25) *			Smokers(*n* = 25) *			Differences Post–Pre IQOS Use between Groups		
	Before	After	*p*	Before	After	*p*	Before	After	*p*	Non-Smokers	Smokers	*p*
**Exhaled CO,** **ppm**	**1.75 ± 1.02**	**4.89 ± 1.4**	**<0.001**	**1.5 ± 1.04**	**4.12 ± 1.66**	**<0.001**	**2.5 ± 1.45**	**4.9 ± 3.6**	**<0.001**	2.6 ± 1.15	2.4 ± 2.3	0.7 ^
**SaO2%**	**98.4 ± 1.2**	**97.9 ± 1.06**	**0.002**	**98.4 ± 1.3**	**97.9 ± 0.9**	**0.04**	**98.4 ± 1.1**	**97.9 ± 1.2**	**0.02**	−1 (−2–2)	0 (−2–1)	0.6 #
**FVC, L**	4.37 ± 1.08	4.37 ± 1.03	0.97	4.37 ± 1.04	4.4 ± 1.02	0.57	4.36 ± 1.14	4.34 ± 1.06	0.55	0.04 (−0.44–0.76)	−0.03 (−0.31–0.36)	0.25 #
**FEV1, L**	3.73 ± 0.92	3.7 ± 0.9	0.22	3.79 ± 0.87	3.76 ± 0.88	0.55	3.67 ± 1	3.64 ± 0.94	0.22	−0.04 (−0.75–0.44)	−0.02 (−0.6–0.21)	0.77 #
**FEV1/FVC** **(%)**	84.36 ± 6.38	83.6 ± 7.57	0.15	85.56 ± 5.76	84.6 ± 7.5	0.2	83.2 ± 6.85	82.6 ± 7.67	0.5	−0.96 ± 3.6	−0.52 ± 3.7	0.67 ^
**FEF 25%,** **L/s**	**7.38 ± 1.9**	**6.98 ± 1.92**	**0.002**	**7.6 ± 1.89**	**7.14 ± 2.06**	**0.009**	7.14 ± 1.93	6.83 ± 1.81	0.08	−0.35 (−2.52–0.83)	−0.14 (−1.69–1.35)	0.52 #
**FEF 50%,** **L/s**	**5.00 ± 1.42**	**4.84 ± 1.45**	**0.03**	5.19 ± 1.43	5.02 ± 1.53	0.09	4.82 ± 1.43	4.68 ± 1.4	0.18	−0.17 ± 0.48	−0.14 ± 0.5	0.8 ^
**FEF75%,** **L/s**	1.92 ± 0.84	1.87 ± 0.88	0.38	2.05 ± 0.81	1.99 ± 0.93	0.58	1.8 ± 0.86	1.75 ± 0.83	0.46	−0.03 (−1.02–1.31)	−0.04 (−0.8–0.52)	0.9 #
**MMEF,** **L/s**	4.3 ± 1.43	4.19 ± 1.47	0.17	4.5 ± 1.43	4.37 ± 1.04	0.3	4.1 ± 1.44	4.01 ± 1.43	0.39	−0.13 ± 0.6	−0.08 ± 0.5	0.8 ^
**PEF, L/s**	**7.9 ± 2.16**	**7.3 ± 2.08**	**<0.001**	**8.22 ± 2.06**	**7.5 ± 2.16**	**0.001**	**7.69 ± 2.26**	**7.12 ± 2.03**	**0.007**	−0.7 ± 1.01	−0.57 ± 0.9	0.6 ^
**FRC, L**	3.51 ± 1.14	3.3 ± 0.95	0.17	3.35 ± 1.06	3.24 ± 1.1	0.6	3.67 ± 1.23	3.36 ± 0.8	0.2	−0.2 (−1.4–3.07)	−0.11 (−3.28–1.69)	0.77 #
**RV, L**	1.6 ± 0.48	1.59 ± 0.46	0.74	1.54 ± 0.43	1.55 ± 0.42	0.9	1.66 ± 0.53	1.64 ± 0.51	0.4	0.01 (−0.81–1.05)	−0.04 (−0.61–0.28)	0.67 #
**ERV, L**	1.4 ± 0.66	1.24 ± 0.55	0.06	1.23 ± 0.51	1.19 ± 0.62	0.7	**1.57 ± 0.76**	**1.23 ± 0.48**	**0.03**	−0.04 ± 0.5	−0.27 ± 0.6	0.16 ^
**TLC, L**	5.8 ± 1.13	5.81 ± 1.11	0.65	5.75 ± 1.19	5.77 ± 1.16	0.7	5.86 ± 1.1	5.86 ± 1.02	0.9	0.00 (−0.61–1.12)	−0.04 (−0.23–0.46)	0.76 #
**DLCO,** mmol/min/kPa	9.05 ± 2.21	8.94 ± 2.18	0.21	9.64 ± 2.06	9.55 ± 2.13	0.4	8.46 ± 2.24	8.33 ± 2.1	0.35	−0.09 ± 0.5	−0.13 ± 0.67	0.8 ^
**KCO,** mmol/min/kPa/L	**1.61 ± 0.26**	**1.58 ± 0.26**	**0.05**	1.74 ± 0.19	1.71 ± 0.2	0.09	1.49 ± 0.27	1.47 ± 0.26	0.3	−0.02 (−0.29–0.09)	−0.02 (−0.24–0.23)	0.7 #

FVC = forced vital capacity, FEV1 = forced expiratory volume in 1 s, FEF, forced expiratory flow, MMEF = maximal midexpi ratory flow; PEF = peak expiratory flow, RV = residual volume, ERV = expiratory reserve volume, TLC = total lung capacity, DLCO = diffusion lung capacity for carbon monoxide, KCO = DLCO/VA. * Paired samples T test, ^ independent samples T test for parametric, or # Mann–Whitney U test for non-parametric variables.

**Table 3:** Acute effects of IQOS on respiratory resistances of all participant, non-smokers and smokers—differences between the measurements before and immediately after IQOS use.

	All Participants (*n* = 50) *			Non Smokers(*n* = 25) *			Smokers(*n* = 25) *			Differences Post–Pre IQOS Use between Groups **		
	Before	After	*p*	Before	After	*p*	Before	After	*p*	Non Smokers	Smokers	*p*
**Z5 Hz** **(kPa/L/s)**	**0.376 ± 0.104**	**0.398 ± 0.109**	**0.045**	0.39 ± 0.12	0.41 ± 0.12	0.15	0.36 ± 0.09	0.38 ± 0.1	0.16	0.00 (−0.07–0.38)	0.01 (−0.07–0.26)	0.96
**R5 Hz** **(kPa/L/s)**	**0.366 ± 0.102**	**0.388 ± 0.109**	**0.04**	0.38 ± 0.11	0.4 ± 0.11	0.14	0.35 ± 0.09	0.37 ± 0.1	0.16	0.01 (−0.06–0.37)	0.01 (−0.07–0.25)	0.97
**R10 Hz** **(kPa/L/s)**	**0.33 ± 0.097**	**0.345 ± 0.097**	**0.04**	0.34 ± 0.11	0.36 ± 0.1	0.15	0.31 ± 0.08	0.33 ± 0.09	0.16	0.01 (−0.05–0.32)	0.01 (−0.06–0.24)	0.95
**R15 Hz** **(kPa/L/s)**	**0.32 ± 0.095**	**0.34 ± 0.095**	**0.038**	0.33 ± 0.105	0.35 ± 0.1	0.16	0.31 ± 0.08	0.33 ± 0.09	0.12	0.01 (−0.06–0.3),	0.01 (−0.05–0.22)	0.78
**R20 Hz** **(kPa/L/s)**	**0.326 ± 0.09**	**0.345 ± 0.09**	**0.018**	0.34 ± 0.1	0.36 ± 0.09	0.09	0.31 ± 0.08	0.33 ± 0.09	0.1	0.01 (−0.06–0.28)	0.01 (−0.05–0.22)	0.96
**R25 Hz** **(kPa/L/s)**	**0.343 ± 0.095**	**0.36 ± 0.09**	**0.017**	0.36 ± 0.1	0.38 ± 0.09	0.08	0.32 ± 0.09	0.34 ± 0.09	0.11	0.01 (−0.05–0.26)	0.01 (−0.05–0.22)	0.76
**R35 Hz** **(kPa/L/s)**	**0.39 ± 0.11**	**0.41 ± 0.10**	**0.011**	0.42 ± 0.12	0.44 ± 0.09	0.11	**0.36 ± 0.11**	**0.39 ± 0.11**	**0.047**	0.00 (−0.07–0.26)	0.02 (−0.06–0.23)	0.66

Z5 Hz = respiratory impedance at 5 Hz, R5 Hz, R10 Hz, R15 Hz, R20 Hz, R25 Hz, R35 Hz = respiratory resistance at 5, 10, 15, 20, 25, 35 Hz. * Paired samples T test. ** Mann–Whitney U test.

**Table 4:** Regression coefficients (β) and confidential interval (CI) 95% for β from linear models for the changes in the measurements of pulmonary function test, impulse oscillometry (IOS) before and immediately after IQOS, unadjusted and adjusted for age and pack/years.

	Unadjusted			Adjusted for Age and Pack/Years		
	β	CI 95%	*p*-Value	β	CI 95%	*p*-Value
**Exhaled CO,** **ppm**	0.2	0.056–1.23	0.69	0.89	0.39–2.18	0.2
**SaO2%**	0.01	−0.59–0.599	0.99	−0.07	−0.86–0.71	0.85
**FVC, L**	−0.047	−0.161–0.067	0.4	−0.048	−0.162–0.067	0.4
**FEV1, L**	−0.013	−0.121–0.096	0.8	0.048	−0.096–0.193	0.5
**FEV1/FVC** **(%)**	0.448	−1.63–2.527	0.67	2.26	−0.49–5.008	0.1
**FEF 25%,** **L/s**	0.182	−0.309–0.67	0.46	0.158	−0.518–0.834	0.64
**FEF 50%,** **L/s**	0.33	−0.247–0.313	0.81	0.1	−0.278–0.486	0.59
**FEF75%,** **L/s**	0.005	−0.228–0.238	0.96	0.072	−0.248–0.392	0.65
**MMEF,** **L/s**	0.044	−0.266–0.353	0.77	0.192	−0.224–0.608	0.36
**PEF, L/s**	0.152	−0.41–0.715	0.59	0.395	−0.37–1.16	0.31
**FRC, L**	−0.212	−0.833–0.41	0.5	0.293	−0.53–1.116	0.48
**RV, L**	−0.033	−0.19–0.124	0.67	−0.075	−0.287–0.137	0.48
**ERV, L**	−0.232	−0.56–0.099	0.16	−0.143	−0.599–0.313	0.53
**TLC, L**	−0.021	−0.159–0.116	0.76	0.064	−0.122–0.25	0.49
**DLCO,** mmol/min/kPa	−0.036	−0.386–0.313	0.83	−0.019	−0.49–0.45	0.94
**KCO,** mmol/min/kPa/L	0.008	−0.043–0.06	0.74	−0.019	−0.089–0.05	0.58
**Z5 Hz** **(kPa/L/s)**	−0.007	−0.05–0.035	0.73	0.003	−0.053–0.06	0.9
**R5 Hz** **(kPa/L/s)**	−0.008	−0.049–0.033	0.69	0.002	−0.053–0.056	0.96
**R10 Hz** **(kPa/L/s)**	−0.005	−0.04–0.032	0.79	0.006	−0.043–0.055	0.81
**R15 Hz** **(kPa/L/s)**	−0.003	−0.038–0.032	0.87	0.007	−0.039–0.054	0.75
**R20 Hz** **(kPa/L/s)**	−0.004	−0.037–0.028	0.78	0.005	−0.038–0.049	0.8
**R25 Hz** **(kPa/L/s)**	−0.005	−0.036–0.027	0.76	0.002	−0.04–0.044	0.92
**R35 Hz** **(kPa/L/s)**	0.002	−0.032–0.035	0.92	0.013	−0.031–0.057	0.56

FVC = Forced vital capacity, FEV1 = forced expiratory volume in 1 s, FEF, forced expiratory flow, MMEF = maximal midexpi ratory flow; PEF = peak expiratory flow, RV = residual volume, ERV = expiratory reserve volume, TLC = total lung capacity, DLCO = diffusion lung capacity for carbon monoxide, KCO = DLCO/VA. Z5 Hz = respiratory impedance at 5 Hz, R5 Hz, R10 Hz, R15 Hz, R20 Hz, R25 Hz, R35 Hz = respiratory resistance at 5, 10, 15, 20, 25, 35 Hz.
